# *Aloe Vera*-Fermented Beverage Ameliorates Obesity and Gut Dysbiosis in High-Fat-Diet Mice

**DOI:** 10.3390/foods11223728

**Published:** 2022-11-20

**Authors:** Shijie Fu, Yanting Dang, Huilin Xu, Aimin Li, Xiaoman Zhou, Xiaodong Gao, Zijie Li

**Affiliations:** 1 Key Laboratory of Carbohydrate Chemistry and Biotechnology, Ministry of Education, School of Biotechnology, Jiangnan University, Wuxi 214122, China; 2 Guozhen Health Technology (Beijing) Co., Ltd., Beijing 102206, China; 3 State Key Laboratory of Biochemical Engineering, Institute of Process Engineering, Chinese Academy of Sciences, Beijing 100190, China

**Keywords:** obesity, fermented beverages, *Aloe vera*, gut microbiota

## Abstract

*Aloe vera* has been proven to have various medicinal properties, including anti-inflammatory and anti-obesity functions. However, the effects of *Aloe vera*-fermented beverages (AFB) on obesity and its complications are still not clear. In this study, HepG2 cells in high-fat environment and high-fat diet (HFD) mice were used to investigate the potential obesity-preventing function of AFB. We found that AFB intervention decreased the amount of lipid droplets of HepG2 cells, suppressed the body weight gain and adipose accumulation, and reduced the serum contents of total cholesterol (TC), alanine aminotransferase (ALT), and interleukin 10 (IL-10) of HFD-mice. In addition, it also changed the composition of the gut microbiota. The ratio of *Firmicutes/Bacteroidetes* was decreased, while the relative abundance of *Muribaculaceae*, *Alistipes* and *Rikenellaceae_RC9_gut_group* was increased after the administration of AFB compared with HFD-mice. These results demonstrated that AFB can prevent diet-induced obesity (DIO) and provides a new option to modulate obesity-related gut dysbiosis.

## 1. Introduction

Obesity, defined as excessive adipose accumulation caused by excess energy intake and insufficient energy expenditure [1], has become an increasingly prevalent disease [2]. Over 70.2% of adults in the United States are overweight or obese according to a survey conducted by the National Health and Nutrition [3]. Additionally, the rate of obesity in children has increased dramatically in recent years [4]. As the current pharmacotherapies are always limited by the adverse effects [5], functional foods with obesity-preventing activity and without adverse effects have great research value.

*Aloe vera*, which belongs to the family Liliaceae, has been proven to have anti-obesity [6] and anti-inflammation functions and is able to modulate blood glucose and cholesterol [7]. Some evidence has indicated that *Aloe vera* gel could alleviate type 2 diabetes, decrease the blood glucose levels [8], and prevent adipose accumulation [9], which might be credited to the phytosterols rich in the gel [10]. It has been proven that *Aloe vera* extracts can also activate brown adipose tissue [11] and suppress the expression of lipogenic genes in mice [12]. Additionally, the milk with the addition of *Aloe vera* gel powder also shows anti-obesity and anti-inflammatory properties after fermentation [13]. Not only milk, but fermented beverages made from some plants were demonstrated to have the function of restraining the body weight gain of mice fed a high-fat diet (HFD) [14,15]. Fermentation can increase the contents [16] and bioavailability [17] of polyphenols and flavonoids in the fermented beverages, which are critical in obesity suppression. Some evidence revealed that fermented beverages could also ameliorate gut microbiota dysbiosis related with HFD [18,19]. Researchers have proven that there are close connections among gut microbiota, obesity, and inflammatory responses [20,21]. The systems that have considerable influences on obesity, including metabolism, energy balance, homeostasis, central appetite, and food-reward signaling, are all regulated by obesity-associated gut microbiota through fermentation in the host [22]. Hence, the *Aloe vera*-fermented beverage (AFB) that the affects obesity and gut microbiota is rarely reported and has high research value.

This study aimed to assess the restorative effects of AFB on the HepG2 cells in a high-fat environment, and the obesity prevention and gut microbiota modulation effects in a HFD C57BL/6J mice model. The body weight, serum, cell morphology of the adipose tissue and liver, gut microbiota, and SCFA in the feces of the mice were examined. Our research may provide a theoretical basis for the development of *Aloe vera*-fermented beverages as a new kind of anti-obesity dietary supplement.

## 2. Materials and Methods

### 2.1. Reagents and Materials

*Aloe vera* (*Aloe vera* (L.) Burm.f.) leaves were provided by Tongxiang Huimei *Aloe* planting Cooperative (Jiaxing, China). *Lactobacillus plantarum* powder was obtained from Guozhen Health Science and Technology (Beijing) Co., Ltd. (Beijing, China). The Princess Fermented beverage (PFB) was purchased from Rivaland Co., Ltd. (Suzuka City, Mie Prefecture, Japan). Folin-Ciocalteu, NaOH, and Al(NO_3_)_3_ were purchased from Sinopharm Chemical Reagent Co., Ltd. (Shanghai, China). Palmitic acid (PA) was from Sigma (St. Louis, MO, USA). Bovine serum albumin (BSA), Dulbecco’s modified Eagle’s medium (DMEM), and fetal bovine serum (FBS) from BBI Solutions (Shanghai, China) were used in cell culture. Cell Counting Kit-8 (CCK-8) was from MedChemExpress (Monmouth, NJ, USA). The 4% paraformaldehyde, Hematoxylin and Eosin Staining Oil (H&E) and Red O kit were purchased from Beyotime (Nantong, China), while the Mayer’s Hematoxylin was from Sbjbio (Nanjing, China). Furthermore, serum interleukin 10 (IL-10) and tumor necrosis factor alpha (TNF-*α*) kits were from Shanghai Enzyme-linked Biotechnology Co., Ltd. (Shanghai, China). The E.Z.N.A. ^®^ soil DNA Kit and the AxyPrep DNA Gel Extraction Kit were from Omega Bio-Tek (Norcross, GA, USA) and Axygen Biosciences (Union City, CA, USA) respectively.

### 2.2. Preparation and Composition of the Fermented Juice

To prepare the AFB, 300 g *Aloe vera* leaves were cut into 8 cm segments and added to 700 mL water before the addition of 100 g brown granulated sugar. After the first step of fermentation that took 3 months at room temperature, 2.2 mg *Lactobacillus plantarum* powder was added for the second step of fermentation, which was maintained at 29 ± 2 °C for 3–7 days. When the pH value was below 4, the fermentation was terminated and the AFB was stored at −20 °C for a maximum of 2 month after sterilization at 105 °C for 30 min. The Princess Fermented beverage (PFB) was used as a positive control. Folin-Ciocalteu and Al(NO_3_)_3_ were used as color developing agents to detect the contents of total polyphenols and flavonoids, respectively. The content of total proteins was measured by the Coomassie Brilliant Blue method. Total sugars and reducing sugars were detected by using anthrone-sulfuric acid and the DNS method, respectively. The compositions of the two kinds of beverages are provided in Table S1.

### 2.3. Preparation of Palmitic Acid and AFB Dilution

Palmitic acid (PA) was prepared according to a reported method [23]. To obtain the stoke PA solution, 1 mL of 100 mM NaOH solution which contained 0.1 mmol PA was mixed with 19 mL of 10% bovine serum albumin (BSA) at 70 °C, then stored at −20 °C after membrane filtration. The stoke PA solution and the AFB were diluted to 150, 200, 250, 300, and 350 μM and 1%, 2%, 3%, 4%, and 5% by Dulbecco’s modified Eagle’s medium (DMEM), respectively.

### 2.4. Cell Culture and Assay

HepG2 cells were cultured in DMEM with 10% fetal bovine serum (FBS) and maintained at 37 °C in a moist atmosphere containing 5% CO_2_. To investigate the cytotoxicity of PA and AFB, the HepG2 cells were exposed to different concentrations of PA (150, 200, 250, 300, and 350 μM) and AFB (1%, 2%, 3%, 4%, and 5%) in 96-well plates for 48 h. The medium was replaced by 100 μL fresh DMEM and 10 μL Cell Counting Kit-8 (CCK-8) before incubation at 37 °C for 1 h. The absorbance was detected by a microplate reader (Bio-Rad Laboratories, Berkeley, CA, USA) under 450 nm to analyze the cell viability. To figure out the restorative effects of AFB on HepG2 cells in the high-fat environment, the cells were cultured by 200 μM PA (PA group), 200 μM PA, and 2% AFB (PA + AFB group), without any PA or AFB (control group) for 48 h, then detected by CCK-8.

### 2.5. Oil Red O Staining

After cultured for 48 h, the cells in 24-well plates from the control, PA, and PA + AFB groups were washed and fixed in 4% paraformaldehyde for 30 min before staining with the Oil Red O kit. The cells were observed under an inverted microscope (Nikon Corporation, Tokyo, Japan) after counterstaining by Mayer’s Hematoxylin.

### 2.6. Animal Experimental Design

Male C57BL/6J mice at 5 weeks old were purchased from Vital River Laboratory Animal Technology Co., Ltd. (Jiaxing, China). The animals were kept in a specific room with a temperature of 23 ± 3 °C, a relative humidity of 55 ± 15% and a controlled 12 h light–dark cycle. After the first week of acclimation, 8 mice were chosen randomly to receive a normal diet as the control group (ND) and the others were given a high-fat diet (HFD) as the HFD0 group. The compositions of the two kinds of diets are provided in Table S2. After two weeks, the weights of the mice in the HFD0 group were measured, and the heaviest 40 mice were chosen randomly divided into 5 different groups (*n* = 8), including the HFD group, the PFB group (HFD + PFB), the LAFB group (HFD + low dose of AFB), the MAFB group (HFD + medium dose of AFB), and the HAFB group (HFD + high dose of AFB). The mice in the LAFB, MAFB, and HAFB groups were given 3.9, 7.8, and 15.6 mL/kg body weight AFB each day, respectively. In the PFB group, the mice were given 7.8 mL/kg body weight PFB every day, while in the ND and HFD groups, the mice were given the same volume of saline. Their body weights were measured once a week. After week 8, fecal samples from the mice were collected and stored at −80 °C. Finally, the mice were fasted for 12 h and then anesthetized with pentobarbital sodium. The blood was collected from an eyeball and centrifuged at 1150 g for 15 min after clotting for 12 h at 4 °C to obtain the serum. In addition, the liver and adipose tissue were weighed and then fixed in 4% paraformaldehyde after washing with PBS. All experimental procedures were examined and approved by the Animal Ethics Committee of Jiangnan University (Permission Number: JN. No20210315c1150610[004]).

### 2.7. Serum Biochemical Analysis

Serum alanine aminotransferase (ALT), aspartate aminotransferase (AST), high-density lipoprotein cholesterol (HDL-C), low-density lipoprotein cholesterol (LDL-C), total cholesterol (TC), and triacylglycerol (TG) were measured by an automatic biochemical analyzer (Shenzhen Mindray Bio-Medical Electronics Co., Ltd., Shenzhen, China). Furthermore, serum interleukin 10 (IL-10) and tumor necrosis factor alpha (TNF-*α*) were measured by commercial kits. Under the instructions of the kits, 50 μL serum was mixed with 100 μL HRP-conjugate reagent in the microtiter plates and incubated for 30 min. Afterwards, the microtiter plates were washed 5 times by the wash solution before 50 μL substrate A and substrate B were added into the microtiter plates. After incubation for 15 min at 37 °C, the stop solution was added into the microtiter plates and the absorbance was measured under 450 nm by a microplate reader (Bio-Rad Laboratories). The blank and standard samples were analyzed following the same instructions in the same time.

### 2.8. Histopathological Examinations

Liver and epididymal adipose tissues were collected and fixed in 4% paraformaldehyde for at least 24 h and then embedded in paraffin. The tissues were sliced into 4 μm sections before staining with hematoxylin-eosin (H&E). An inverted microscope (Nikon Corporation) was used for histological assessment of the tissue sections.

### 2.9. Analysis of Short-Chain Fatty Acids (SCFA)

Approximately 20 mg of fecal sample was added to 500 μL of saturated NaCl for 30 min. After homogenization, 20 μL of H_2_SO_4_ (1.67 mol/L) was added to the mixture. Afterward, 1 mL anhydrous ether was added, mixed for 30 s, allowed to stand for 30 min, and then centrifuged at 4600× *g* for 15 min. The supernatants of the samples were dried by 0.25 g anhydrous Na_2_SO_4_ before injection into a gas chromatograph (GC) with a flame ionization detector 7890 (Agilent Technologies, Inc., Palo Alto, CA, USA) and a fused silica capillary column (Zebron, ZB-FFAP, 30 m × 0.25 mm × 0.25 μm). The contents of SCFA were measured following a reported method [24]. The initial temperature of the oven was raised from 80 °C to 190 °C at a rate of 8 °C/min and kept at 192 °C for 3 min, while the temperature of the injector and detector was 230 °C. Nitrogen that passed through the column at a constant flow rate of 1 mL/min was used as the carrier gas. Quantification of acetic acid, propionic acid, butyric acid, and isobutyric acid was based on the standard solution and relative peak areas from the GC.

### 2.10. Fecal DNA Extraction and Gut Microbiota Analysis

DNA was extracted with the E.Z.N.A. ^®^ soil DNA Kit from feces samples stored at −80 °C. The concentration and purification of the DNA were determined by a NanoDrop 2000 UV–vis spectrophotometer (Thermo Scientific, Waltham, MA, USA). The 16S rRNA gene from the V3-V4 hypervariable regions of the bacteria was amplified with primers 338F (5′-ACTCCTACGGGAGGCAGCAG-3′) and 806R (5′-GGACTACHVGGGTWTCTAAT-3′) by a GeneAmp 9700 PCR system (Applied Biosystems, San Diego, CA, USA). The products were purified by an AxyPrep DNA Gel Extraction Kit (Axygen Biosciences, Union City, CA, USA) after extraction from a 2% agarose gel and further quantified by a Quanti Fluor™-ST (Promega, Madison, WI, USA). The amplicons were then pooled in equimolar amounts and paired-end sequenced (2 × 300) on an Illumina MiSeq platform (Illumina, San Diego, CA, USA). Raw fastq files were quality-filtered by Trimmomatic and merged by FLASH with the following criteria: (i) the reads were truncated at any site receiving an average quality score <20 over a 50 bp sliding window. (ii) Sequences whose overlap was longer than 10 bp were merged according to their overlap with a mismatch of no more than 2 bp. (iii) Sequences of each sample were separated according to the barcodes (exactly matching) and primers (allowing 2 nucleotide mismatches), and reads containing ambiguous bases were removed. Operational taxonomic units (OTU) were clustered with a 97% similarity cutoff using UPARSE 7.1 (http://drive5.com/uparse/, accessed on 10 August 2021) with a novel ‘greedy’ algorithm that performs chimera filtering and OTU clustering simultaneously. The taxonomy of each 16S rRNA gene sequence was analyzed by the RDP Classifier algorithm (http://rdp.cme.msu.edu/, accessed on 10 August 2021) against the Silva (SSU123) 16S rRNA database using a confidence threshold of 70%.

### 2.11. Statistical Analysis

Statistical significance was determined by using GraphPad Prism 8.0 (GraphPad Software, Inc., San Diego, CA, USA) according to one-way ANOVA with Dunnett’s multiple comparisons test. The differences were considered statistically significant when *p* < 0.05 or *p* < 0.01. The results are expressed as mean ± standard deviation (SD).

## 3. Results

### 3.1. The Restorative Effects of AFB on HepG2 Cells in High-Fat Environment

**Figure 1:**
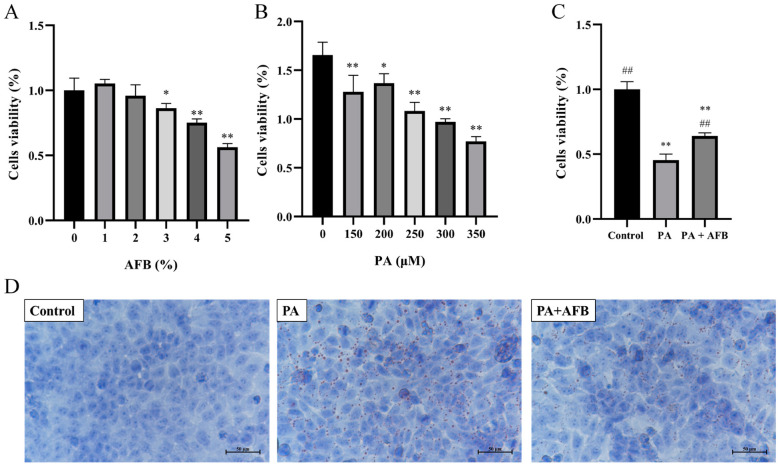
Effect of PA and AFB on the HepG2 cells in the high-fat environment. (**A**) The cell viability after treatments with different concentrations of AFB. (**B**) The cell viability after treatments with different concentrations of PA. (**C**) The cell viability after the treatment of PA or PA + AFB. (**D**) Oil Red O staining of HepG2 cells after treatments with PA or PA + AFB (100×). The results are expressed as the mean ± SD. * *p* < 0.05, ** *p* < 0.01 versus the control group and ^##^ *p* < 0.01 versus the PA group by one-way ANOVA.

In order to investigate the effect of AFB on PA and HepG2 cells, they were exposed to different concentrations of PA and AFB. The cell viability was not affected after the treatment with 2% AFB. However, the cell viability significantly decreased after the treatment with 3–5% AFB (Figure 1A). The treatment of PA at 150–350 μM for 48 h decreased the cell viability significantly while the cells had the highest viability when the concentration of PA was 200 μM (Figure 1B). Based on the results above, 2% AFB and 200 μM PA were selected for the next experiments. When the cells were exposed to the PA and AFB together, the cell viability was significantly higher than PA group and lower than the control group, which indicated that AFB treatment alleviated the damage of HepG2 cells induced by PA (Figure 1C). The results of Oil Red O staining indicated the observed oil droplets induced by the high-fat environment were decreased after AFB treatment (Figure 1D).

### 3.2. AFB Prevented HFD-Induced Obesity

**Figure 2:**
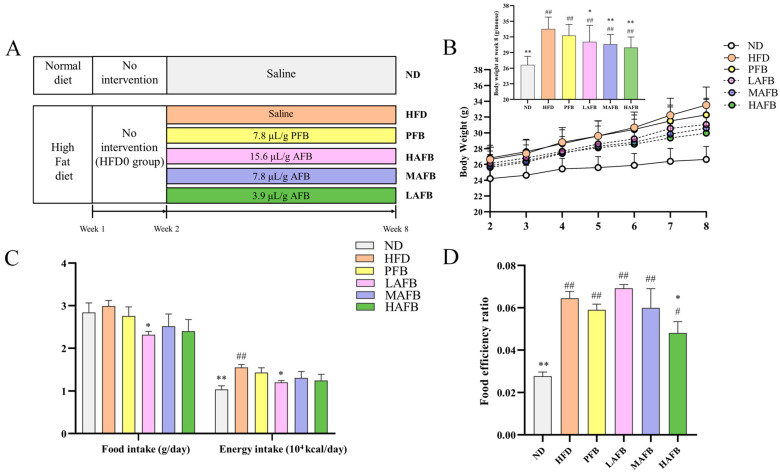
Effect of AFB on body weight, food intake, and food efficiency. (**A**) Schematic diagram of the experiment. (**B**) Growth curve of body weight from week 2 to week 8. (**C**) Average food and energy intake from week 2 to week 8. (**D**) Food efficiency = body weight gain/food intake. The results are expressed as the mean ± SD. * *p* < 0.05, ** *p* < 0.01 versus the HFD group and ^#^ *p* < 0.05, ^##^ *p* < 0.01 versus the ND group by one-way ANOVA.

Different doses of AFB were given to the mice under HFD (Figure 2A). The body weights of the mice were recorded every week during the experiment (Figure 2B). There were no significant differences in the body weights of the mice among the HFD, PFB, LAFB, MAFB, and HAFB groups at week 2. From week 2 to week 8, the body weights of the mice in each group continued to increase, while the body weights of the HFD group were significantly higher than those of the ND group at week 8. After treatment with PFB and AFB, the body weights of the LAFB, MAFB, and HAFB groups were significantly lower than those of the HFD group at week 8. Besides, the food intake and energy intake of mice in the LAFB, MAFB, and HAFB groups were also lower than those in the HFD group (Figure 2C). Although the mice in the LAFB group had lower food intake and energy intake than those in the MAFB and HAFB groups, their higher food efficiency (Figure 2D) might have caused their higher body weights at week 8.

### 3.3. Effect of AFB on Liver and Adipose Tissue

**Figure 3:**
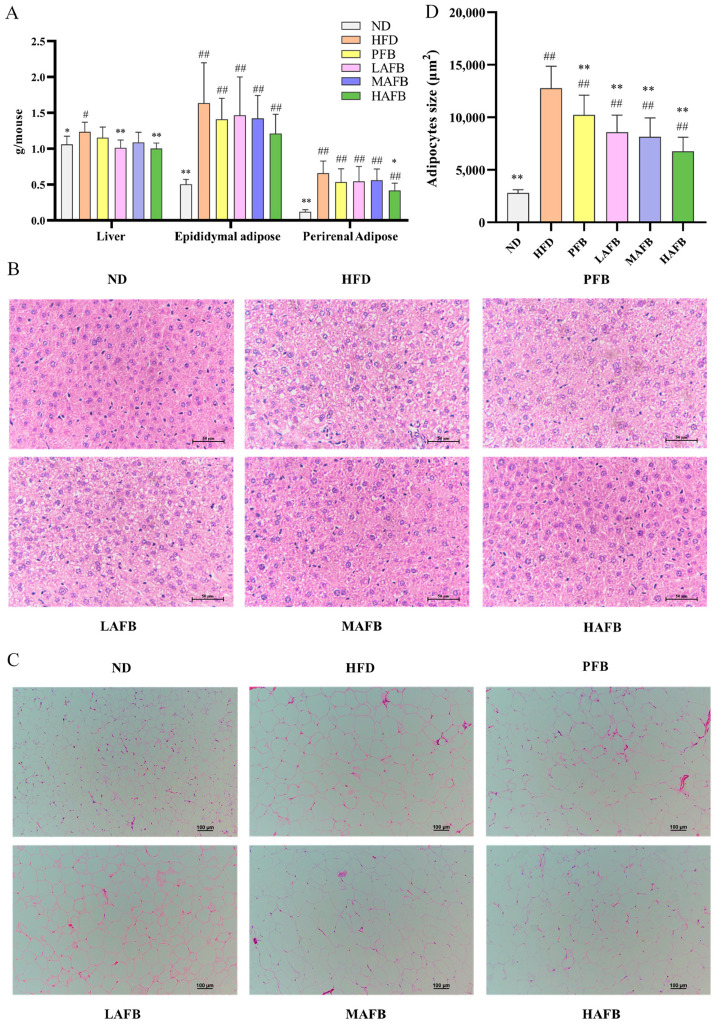
Effect of AFB on tissue. (**A**) Weight of the liver, epididymal adipose, and perirenal adipose tissue. (**B**) H&E staining of the liver (400×). (**C**) H&E staining of epididymal adipose tissue (100×). (**D**) The size of the epididymal adipose tissue. The results are expressed as the mean ± SD. * *p* < 0.05, ** *p* < 0.01 versus the HFD group and ^#^ *p* < 0.05, ^##^ *p* < 0.01 versus the ND group by one-way ANOVA.

After the mice were sacrificed, the liver, perirenal adipose tissue, and epididymal adipose tissue were collected and weighed (Figure 3A). Long-term HFD consumption led to increased liver, epididymal adipose, and perirenal adipose weights in mice compared with those in the ND group. After the administration of AFB, the weights of the three tissues all showed downward trends. In particular, the mice in the HAFB group exhibited significantly lower weights of the livers and perirenal adipose tissue than those in the HFD group. Similar results were also observed in the pathological sections. In the ND group, the liver cells had clear borders and almost no lipid droplets, while the HFD and PFB groups exhibited serious fatty infiltration. However, after AFB intervention, the fatty infiltration was alleviated compared with that in the HFD group (Figure 3B). In addition, the sizes of the epididymal adipose cells in the three AFB-supplemented groups were smaller than those in the HFD group (Figure 3C,D), while the latter were much larger than the cells in the ND group.

### 3.4. Effect of AFB on Serum Biochemical Parameters

ALT and AST are important indicators to assess the extent of liver damage. The AFB-treated groups had significantly lower ALT levels than the HFD group (Table 1). Besides, the TC, TG, HDL-C, and LDL-C levels are important parameters for predicting the risks of hyperlipidemia, cardiovascular and cerebrovascular diseases induced by an HFD. The results reveal that HAFB could significantly decrease TC levels compared with the HFD group. Furthermore, AFB treatment also decreased the TG, HDL-C, LDL-C, and AST levels compared with the HFD group, although the differences were not significant (Table 1). To evaluate the effect of AFB on inflammation, the levels of serum TNF-*α* and IL-10 were also measured. The levels of IL-10 were significantly decreased in the PFB and AFB-supplemented groups, while the levels of TNF-*α* were also decreased (Table 1).

### 3.5. Effect of AFB Intervention on the α and β Diversities of the Gut Microbiota

**Figure 4:**
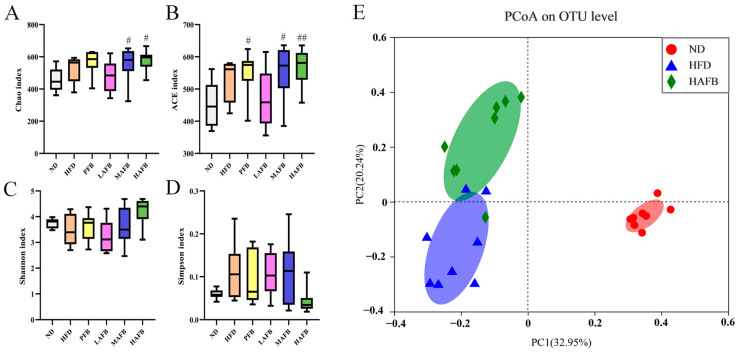
AFB affected the α and β diversity of the mouse gut microbiota. (**A**) Chao index, (**B**) ACE index, (**C**) Shannon index, (**D**) Simpson index, and (**E**) UniFrac distance-based principal coordinate analysis (PCoA) of the ND, HFD, and HAFB groups. ^#^ *p* < 0.05, ^##^ *p* < 0.01 versus the ND group by one-way ANOVA.

Chao, ACE, Shannon, and Simpson indices indicated that the richness and diversities of gut microbiota respectively are used to analyze the *α* diversity. As shown in Figure 4A,B, the Chao and ACE indices of the MAFB and HAFB groups were significantly higher than those in the ND group and slightly higher than those in the HFD group, but the difference was not significant. Similarly, the Shannon index of the HAFB group was higher than those of the ND and HFD groups while the Simpson index was lower, although the differences were not significant (Figure 4C,D).

In addition, PCoA was used to analyze the *β* diversities among the groups. As displayed in Figure 4E, the ND group was obviously separated from the HFD group, indicating that HFD significantly affected the composition of the gut microbiota. Moreover, the HAFB group was almost completely separated from the HFD group, which demonstrated that the supplementation with HAFB significantly changed the structure of gut microbiota of HFD mice. The LAFB and MAFB groups also showed a tendency to be separated from the HFD group and they gradually became closer to the HAFB group with increasing doses of AFB (Figure S1).

### 3.6. Effect of HFD and AFB on the Composition of the Gut Microbiota

**Figure 5:**
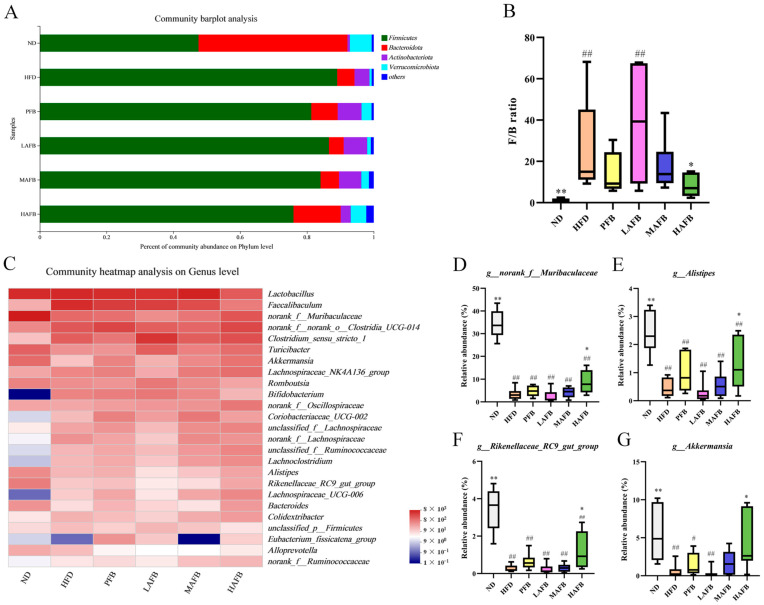
AFB restructured the mouse gut microbiota. (**A**) Relative abundance of gut microbiota at the phylum level. (**B**) F/B ratio. (**C**) Heatmap of the relative abundance of each group at the genus level. (**D**–**G**) The relative abundances of some genera were significantly changed due to the consumption of AFB. * *p* < 0.05, ** *p* < 0.01 versus the HFD group and ^#^ *p* < 0.05, ^##^ *p* < 0.01 versus the ND group by one-way ANOVA.

The intestinal microbiota were mainly composed of *Firmicutes*, *Bacteroidota*, *Actinobacteriota* and *Verrucomicrobiota* at the phylum level (Figure 5A). Compared with the ND group, the relative abundance of *Firmicutes* and *Actinobacteriota* in the HFD group increased, while those of *Bacteroidota* and *Verrucomicrobiota* decreased. In the LAFB, MAFB, and HAFB groups, the relative abundance of *Firmicutes* and *Actinobacteriota* decreased gradually, while the relative abundance of *Bacteroidota* and *Verrucomicrobiota* increased gradually. Besides, the F/B ratio (*Firmicutes* to *Bacteroidetes* ratio) also changed significantly after the intervention of high-dose AFB (Figure 5B). The significantly higher F/B ratio induced by HFD was reversed by high-dose HAFB treatment and led to a significantly lower F/B ratio than that in the HFD group.

At the genus level, the 25 genera with the highest relative abundance were selected to evaluate the effect of AFB on changing the gut microbiome structure of the mice (Figure 5C). *Norank_f__Muribaculaceae*, *Akkermansia*, *Alistipes*, *Rikenellaceae_RC9_gut_group* (Figure 5D–G), and *Bacteroides* were enriched in the ND group and less enriched in the HFD group. After AFB treatment, however, the relative abundance of these genera displayed a dose-dependent increase. Furthermore, HFD induced a higher relative abundance of *Faecalibaculum*, *Bifidobacterium*, and *unclassified_p__Firmicutes* compared with the ND group. After the administration of AFB, the relative abundance of these genera displayed a dose-dependent decrease. Moreover, the relative abundance of *Lachnospiraceae_NK4A136_group*, *unclassified_f__Lachnospiraceae*, *norank_f__Lachnospiraceae*, *Ruminococcaceae*, *Lachnoclostridium*, *Lachnospiraceae_UCG-006*, *Colidextribacter*, and *norank_f__Ruminococcaceae* was also increased after AFB intervention. Moreover, the levels of SCFA also changed after supplementation with AFB (Table 2). The levels of all kinds of SCFA were decreased in the long-term HFD group compared with the ND group, while they were increased after AFB treatment compared with the HFD group, although the difference was not significant.

### 3.7. Obesity and SCFA Levels Were Correlated with the Gut Microbiota

**Figure 6:**
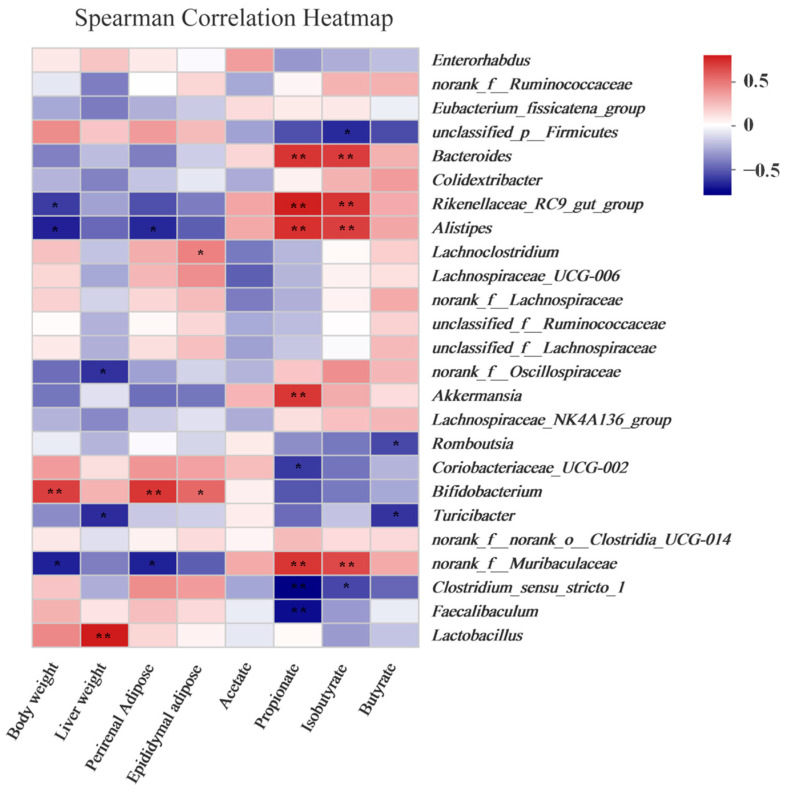
Correlation analysis. The heatmap shows Spearman correlations among the obesity-related parameters and gut microbiota. Spearman correlations among the obesity-related parameters and gut microbiota. Significant correlations are displayed as * *p* < 0.05, ** *p* < 0.01.

Spearman’s correlation analysis was applied to elucidate the potential connections among the gut microbiota and obesity-related indicators. As shown in Figure 6, *Bifidobacterium* showed significantly positive correlations with tissues and body weight and significantly negative correlations with SCFA levels. In contrast, *norank_f__Muribaculaceae, Alistipes*, and *Rikenellaceae_RC9_gut_group* were negatively correlated with body weight and perirenal adipose tissue and positively correlated with propionate and isobutyrate levels. Moreover, after the high dose of AFB treatment, the relative abundance of *norank_f__Muribaculaceae, Alistipes*, and *Rikenellaceae_RC9_gut_group* increased significantly (Figure 5D–F).

## 4. Discussion

With the gradually increasing intake of high-calorie food, obesity has become a global epidemic that places an excessive health burden on humans [25]. Excessive energy intake always induces excessive lipogenesis and dyslipidemia, and then leads to cardiovascular disease [26,27] and inflammatory responses [28]. In addition, excessive lipogenesis also increases the risk of non-alcoholic fatty liver disease [29]. Therefore, finding an ideal solution for this social problem is urgently needed. It has been proved that *Aloe vera* can reduce adipose accumulation [30], alleviate metabolic syndrome [31], and has no reported side effects [32]. Furthermore, some research also indicated that fermented beverages could prevent diet-induced obesity [33]. This study focused on the bioactivities of *Aloe vera*-fermented beverages, which remain underexplored.

The development of obesity stems from the food efficiency ratio, which represents the ability of animals to convert food into body weight gain on a per-gram basis [34]. In our study, the mice in HFD group had significantly higher food efficiency rates than the ND group and HAFB group (Figure 2D), which demonstrated that the intervention of AFB prevented the HFD-mice from excessive energy absorption. By comparing with PFB, we found that AFB had higher contents in polyphenols and flavonoids (Table S1). It has been proven that some of the polyphenols can inhibit the appetite [35], which might contribute to the lower food intake after AFB treatment (Figure 2C). In addition, polyphenols and flavonoids have been proven to prevent obesity by inducing the browning of white adipose tissue (WAT) and regulating the expression of factors such as C/EBP*α*, SREBP-1C, ACC, and FASN, which relate to the synthesis of fatty acids [36]. Therefore, the high contents of these ingredients might lead to a decreased amount of lipid droplets in HepG2 cells (Figure 1D) and lower body weights after the administration of AFB for 6 weeks (Figure 2B). Moreover, the decreased weight of perirenal adipose tissue and liver (Figure 3A), the reduced size of epididymal adipose cells (Figure 3C,D) and the lower level of TC (Table 1) after the treatment of AFB may relate to the high contents of polyphenols and flavonoids. As fat can be synthesized in the liver but cannot be stored, excessive fat synthesis will cause fatty infiltration and liver damage [37]. The relief of fatty infiltration (Figure 3B) and ALT levels (Table 1) after administration of AFB should also be attributed to the high contents of polyphenols and flavonoids that inhibit the fat synthesis. Furthermore, it has been proven that polyphenols and flavonoids possess anti-oxidant properties and can activate the antioxidant pathways to exert anti-inflammatory effects [38,39], which might relate to the decrease of IL-10 level (Table 1).

As reported in previous studies, because of the limitation of digestion, most polyphenols and flavonoids could pass through the small intestine and reach the large intestine instead of entering the blood [40] which might finally ameliorate obesity-related gut dysbiosis. In the present study, the gut microbial communities also showed dose-dependent differences after the administration of AFB (Figure 4E, Figure S1 and Figure 5A). The F/B ratio in HAFB group was significantly lower than that in HFD group. It has been proven that *Firmicutes* contains far more metabolism-related genes compared with *Bacteroidetes* [41]. A high F/B ratio, which makes the organism obtain more energy from its diet [42], is regarded as one of the characteristics of obesity [43]. Therefore, the decreased F/B ratio caused by the consumption of AFB (Figure 5B) might relate to the decreased food efficiency ratio and play an important role in obesity suppression.

From another perspective, the change in gut microbiota at the genus level (Figure 5C–G) further elucidated the anti-obesity mechanism of AFB. Accumulating evidence has indicated that an HFD could decrease the relative abundance of *Muribaculaceae,* which had a positive relationship with the SCFA contents and a negative relationship with body weight gain, weight of the perirenal adipose tissue, and TC and TG levels [44]. In our present work, the relative abundance of *Muribaculaceae* was increased after AFB treatment (Figure 5D), and it was negatively correlated with body weight and perirenal adipose tissue (Figure 6), which is consistent with previous studies. *Alistipes* has a negative relationship with body, liver, perirenal, and epididymal adipose weight [45]. Some scholars verified that *Alistipes* had a positive relationship with SCFA and could alleviate HFD-induced obesity and its complication by fecal microbiota transplantation [46]. The relative abundance of *Alistipes* was also increased after supplementation in the present study (Figure 5E and Figure 6) which might exert critical effect on obesity prevention. In addition, the evidence suggested that the *Rikenellaceae_RC9_gut_group*, which had a lower relative abundance in obese individuals, was negatively correlated with serum lipids, glucose, and insulin level [47] and positively correlated with SCFA [48]. In our present work, the *Rikenellaceae_RC9_gut_group* exhibited similar characteristics, while its relative abundance was increased after the consumption of AFB (Figure 5F and Figure 6). It was verified that SCFA can induce the browning of white adipose tissue and increase the expression of peroxisome proliferator-activated receptor-*γ* coactivator 1*α* (PGC1*α*) and uncoupling protein 1 (UCP1), which activate energy expenditure and lipid oxidation, thereby causing the loss of body weight [49,50]. Besides, SCFA also suppresses appetite and energy intake through central nervous system-related mechanisms and the gut–brain axis [51]. In sum, the AFB intervention changed the structure of gut microbiota, enhanced the relative abundance of beneficial genera, and finally alleviated the pathological development of metabolic disorders.

## 5. Conclusions

Our research indicated that AFB intervention alleviated lipid accumulation and the complications of obesity, including dyslipidemia, inflammatory responses, and liver damage. In addition, the administration of AFB also changed the structure of the gut microbiota. The F/B ratio was significantly decreased and the relative abundance of beneficial gut microbiota including *Muribaculaceae*, *Alistipes*, and *Rikenellaceae_RC9_gut_group* were significantly increased. Moreover, these three genera of gut microbiota, which might exert critical effects in obesity prevention, showed significantly positive relationships with body weight and perirenal adipose. These results demonstrated that AFB has the potential to become a healthy beverage to prevent obesity.

## Figures and Tables

**Table 1:** Effect of AFB administration on serum parameters.

	ND	HFD	PFB	LAFB	MAFB	HAFB
TC (mmol/L)	2.54 ± 0.18 **	5.08 ± 0.07 ^##^	4.71 ± 0.54 ^##^	4.83 ± 0.23 ^##^	4.61 ± 0.39 ^##^	4.30 ± 0.34 ^##,^**
TG (mmol/L)	0.92 ± 0.08	1.00 ± 0.31	0.96 ± 0.11	0.86 ± 0.15	0.77 ± 0.19	0.74 ± 0.07
HDL-C (mmol/L)	2.10 ± 0.18 **	3.96 ± 0.15 ^##^	3.59 ± 0.41 ^##^	3.76 ± 0.12 ^##^	3.67 ± 0.10 ^##^	3.58 ± 0.26 ^##^
LDL-C (mmol/L)	0.30 ± 0.06 **	0.79 ± 0.30 ^##^	0.87 ± 0.19 ^##^	0.78 ± 0.11 ^##^	0.75 ± 0.03 ^##^	0.59 ± 0.10 ^#^
ALT (U/L)	497.25 ± 5.38	514.30 ± 21.31	473.22 ± 21.23 **	478.82 ± 16.57 *	472.78 ± 4.98 **	477.06 ± 20.16 **
AST (U/L)	103.98 ± 10.92	107.76 ± 23.19	101.30 ± 14.46	91.56 ± 12.15	106.66 ± 18.54	90.98 ± 17.52
TNF-*α* (pg/mL)	17.94 ± 9.19	24.26 ± 10.37	21.02 ± 9.05	20.11 ± 13.95	17.09 ± 7.42	15.53 ± 7.51
IL-10 (pg/mL)	52.81 ± 34.82 **	215.01 ± 12.55 ^##^	75.78 ± 17.98 **	66.7 ± 42.56 **	54.94 ± 38.16 **	40.6 ± 40.07 **

* The results were expressed as mean ± SD. ND: normal diet group; HFD: high-fat diet group; PFB: HFD + 7.8 mL/kg PFB; LAFB: HFD + 3.9 mL/kg AFB; MAFB: HFD + 7.8 mL/kg of AFB; HAFB: HFD + high dose of 15.6 mL/kg AFB. * *p* < 0.05, ** *p* < 0.01 versus HFD group and ^#^ *p* < 0.05, ^##^ *p* < 0.01 versus ND group by one-way ANOVA examination.

**Table 2:** Concentration of SCFA in the feces of each group of mice.

	ND	HFD	PFB	LAFB	MAFB	HAFB
Acetate (μg/g)	1076.92 ± 381.12	510.78 ± 129.98	656.79 ± 248.34	661.93 ± 416.96	660.58 ± 474.07	618.82 ± 113.93
Propionate (μg/g)	687.00 ± 266.87 **	292.89 ± 101.46 ^##^	184.74 ± 89.85 ^##^	105.7 ± 45.75 ^##^	223.83 ± 102.67 ^##^	255.66 ± 108.68 ^##^
Butyrate (μg/g)	185.29 ± 25.62	171.71 ± 51.1	149.61 ± 68.19	182.42 ± 107.22	207.29 ± 130.05	331.67 ± 252.53
Isobutyrate (μg/g)	164.98 ± 45.65	146.31 ± 40.23	84.98 ± 1.49	81.93 ± 51.63	124.35 ± 73.32	215.71 ± 93.39
Total SCFA (μg/g)	1588.55 ± 794.69	1171.43 ± 169.26	1306.05 ± 575.37	976.21 ± 606.54	1097.47 ± 732.45	1453.19 ± 926.13

The results were expressed as mean ± SD, *n* = 8. ND: normal diet group; HFD: high-fat diet group; PFB: HFD + 7.8 mL/kg PFB; LAFB: HFD + 3.9 mL/kg AFB; MAFB: HFD + 7.8 mL/kg of AFB; HAFB: HFD + high dose of 15.6 mL/kg AFB. ** *p* < 0.01 versus HFD group and ^##^ *p* < 0.01 versus ND group by one-way ANOVA examination.

## Data Availability

All data presented within the article is available upon reasonable request from the corresponding authors.

## Supplementary Materials

The following supporting information can be downloaded at: https://www.mdpi.com/article/10.3390/foods11223728/s1, Figure S1: UniFrac distance-based principal coordinate analysis (PCoA) of all 6 groups; Table S1: The compositions of two kinds of beverages; Table S2: The contents of different ingredients in different diet (% of diet, *w*/*w*).
