# Supplementary material for: Aloe Vera-Fermented Beverage Ameliorates Obesity and Gut Dysbiosis in High-Fat-Diet Mice
Source: Foods. 2022 Nov 20;11(22):3728. doi: 10.3390/foods11223728 (PMC9689851; doi:10.3390/foods11223728)
Supplement: Supplementary file 1 [file foods-11-03728-s001.zip › foods-2008824-supplementary.pdf]

Supporting materials

Table S1. The compositions of two kinds of beverages.

Chemical substances	PFB	AFB
Total polyphenols (mg/mL)	7.57±0.28	91.23±2.69
Total protein (mg/mL)	0.27±0.02	0.19±0.00
Total flavonoids (mg/mL)	0.11±0.02	0.24±0.02
Total sugars (mg/mL)	64.07±3.96	444.86±13.99
Total reducing sugars (mg/mL)	6.9±0.06	181.82±3.96

Table S2. The contents of different ingredients in different diet (% of diet, *w/w*).

Ingredients	ND (%)	HFD (%)
corn	0.48	-
wheat middlings	0.12	-
wheat	0.17	-
Soybean meal	0.07	-
Peruvian Fish Meal	0.04	-
American Chicken Meal	0.05	-
animal premix	0.04	-
Stone powder	0.01	-
Soybean oil	0.01	-
Normal diet	-	0.40
Lard	-	0.28
Sucrose	-	0.1
Whole milk power	-	0.08
Casein	-	0.02
Calcium bicarbonate	-	0.02

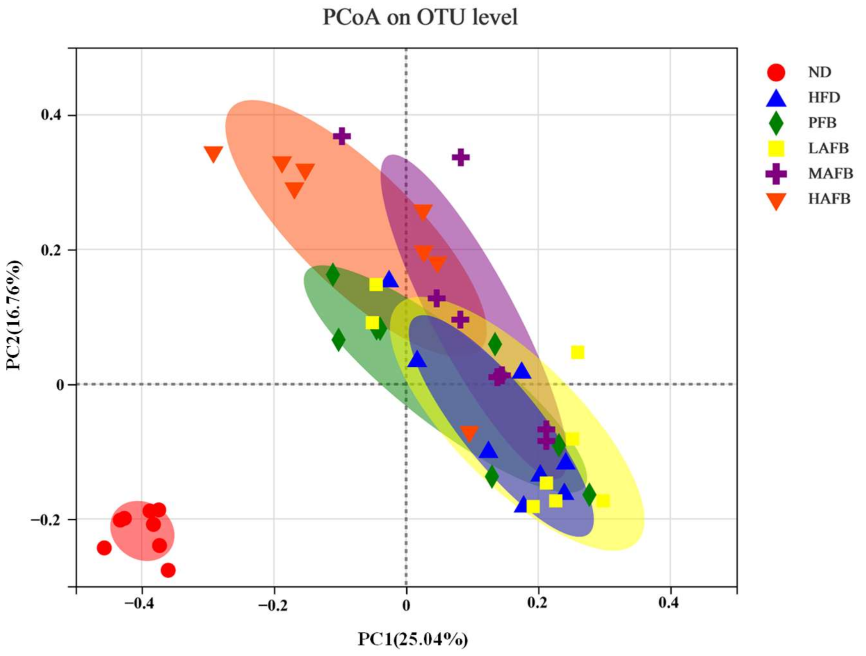

Figure S1. UniFrac distance-based principal coordinate analysis (PCoA) of all 6 groups.
